# Inhibition of vascular endothelial growth factor‐A downregulates angiogenesis in psoriasis: A pilot study

**DOI:** 10.1002/ski2.245

**Published:** 2023-05-15

**Authors:** Andrea Luengas‐Martinez, Dina Ismail, Ralf Paus, Helen S. Young

**Affiliations:** ^1^ Centre for Dermatology Research and Manchester Academic Health Science Centre The University of Manchester Manchester UK; ^2^ Dr. Philip Frost Department of Dermatology and Cutaneous Surgery University of Miami Miller School of Medicine Miami Florida USA; ^3^ Monasterium Laboratory Muenster Germany; ^4^ CUTANEON Hamburg Germany

## Abstract

**Background:**

Vascular Endothelial Growth Factor (VEGF)‐A‐mediated angiogenesis participates in the pathogenesis of psoriasis, thus inviting the hypothesis that anti‐VEGF‐A therapy could be beneficial in psoriasis. While anti‐angiogenic agents are used in oncology and ophthalmology, these therapeutic strategies remain unexplored for the management of psoriasis.

**Objective:**

Our objective was to investigate ex vivo how VEGF‐A blockade impacts blood vessels, epidermis and immune cells in organ‐cultured plaque and non‐lesional skin from patients with psoriasis.

**Methods:**

Skin biopsies from patients with psoriasis (*n* = 6; plaque and non‐lesional skin) and healthy controls (*n* = 6) were incubated with anti‐VEGF‐A monoclonal antibody (bevacizumab, Avastin®) or a human IgG_1_ isotype control for 72‐h in serum‐free organ culture. CD31/LYVE‐1, Ki‐67, and mast cell tryptase expression were assessed by quantitative immunohistomorphometry. VEGF‐A levels in plasma, PBMCs and skin culture supernatants were measured.

**Results:**

Inhibition of VEGF‐A blocked all free VEGF‐A ex vivo, reduced blood vessel area and the number of blood vessel endothelial cells in plaques of psoriasis (*p < 0.05). The treatment effect correlated significantly with levels of VEGF‐A in organ culture supernatants (*r* = 0.94; *p < 0.05) from plaque skin and with plasma levels of VEGF‐A from patients with psoriasis (*r* = 0.943; *p = 0.017).

**Conclusions:**

These ex vivo data are the first studies to objectively investigate the potential of VEGF‐A inhibition as a novel adjuvant treatment strategy for psoriasis. Taken together, our data encourage further investigation by clinical trial to explore whether downregulating pathological angiogenesis has clinical utility, especially in patients with severe psoriasis or those with elevated levels of VEGF‐A in plasma and/or skin.

## Graphical abstract

We report that vascular endothelial growth factor‐A (VEGF‐A) inhibition downregulates inflammatory angiogenesis in psoriasis plaque skin ex vivo. Our data suggest that anti‐angiogenic agents, which are used in oncology and ophthalmology, could be repurposed and integrated into the management of patients with psoriasis. We also explore whether patients with severe psoriasis and/or high levels of VEGF‐A in skin/plasma could benefit most from VEGF‐A blocking therapy.

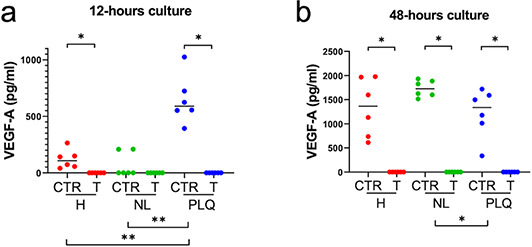

**What is already known about this topic?**

- Vascular endothelial growth factor‐A (VEGF‐A)‐ mediated angiogenesis participates in the pathogenesis of psoriasis.

**What does this study add?**

- Vascular endothelial growth factor‐A (VEGF‐A) inhibition downregulates blood vessel area in psoriasis plaque skin ex vivo.
- Downregulation of blood vessel area is more abrupt in patients with high levels of VEGF‐A and severe disease.

**What is the translational message?**

- This raises the question whether patients with severe psoriasis and/or high levels of VEGF‐A in skin/plasma could benefit most from VEGF‐A blocking therapy.

## 1 INTRODUCTION

Psoriasis is an immune‐mediated disease,^1^ affecting 2%–4% of the world's population.^2^ It is associated with reduced quality of life^3^ and a higher risk of comorbidities such as cardiovascular disease,^4^ diabetes and metabolic syndrome.^5^, ^6^ Aberrant angiogenesis, a feature of the disease pathology, plays a more important role in psoriasis pathogenesis than is widely appreciated.^7^, ^8^, ^9^ Vascular abnormalities precede epidermal hyperplasia in the development of lesions of psoriasis^10^, ^11^ and the clearance of lesions of psoriasis during therapy is accompanied by vascular normalisation.^12^ Vascular abnormalities are macroscopically visible and represent the Auspitz phenomenon, a characteristic feature of psoriasis.^13^

Cutaneous angiogenesis in patients with psoriasis is characterised by pronounced vascular dilation and elongation, in conjunction with enhanced vascular permeability.^14^, ^15^ Vascular endothelial growth factor‐A (VEGF‐A), a potent pro‐angiogenic cytokine, is highly expressed in the skin and plasma of patients with psoriasis,^16^, ^17^ promoting endothelial cell (EC) proliferation, migration, survival, increased vasodilation and permeability^9^, ^18^; as well as keratinocyte proliferation and differentiation.^19^, ^20^ Two common single nucleotide polymorphisms (SNPs) of the VEGF‐A gene, at −460 (rs833061) and +405 (rs2010963), determine VEGF‐A production, both in health and disease.^21^, ^22^ Moreover, individuals who are ‘high VEGF‐A producers’, are significantly more likely to develop psoriasis and to manifest a severe psoriasis phenotype.^21^

Despite preclinical evidence^23^, ^24^, ^25^, ^26^, ^27^, ^28^ and substantial evidence from case reports of resolution of psoriasis whilst on treatment with anti‐VEGF‐A therapies,^29^, ^30^, ^31^, ^32^, ^33^, ^34^, ^35^ the effects of VEGF‐A inhibition remain unexplored in psoriasis, thus raising the question of whether anti‐VEGF‐A therapy could be integrated into psoriasis management by repurposing licenced anti‐VEGF‐A biologicals. Therefore, we postulated that VEGF‐A inhibition represents a novel adjuvant treatment strategy for psoriasis, particularly in patients who are ‘high VEGF‐A producers’.^36^

We interrogated this hypothesis by exposing organ‐cultured plaque and non‐lesional psoriatic skin to treatment with bevacizumab (Avastin®, Roche, Basel, Switzerland), a humanised monoclonal antibody against VEGF‐A or isotype control IgG_1_ ex vivo.^15^, ^37^ The effects of bevacizumab ex vivo were assessed by quantitative immunohistomorphometry on blood vessels using double immunofluorescence staining CD31/LYVE‐1 (CD31 is an endothelial cell marker and LYVE‐1 is a lymphatic‐specific endothelial cell marker) to visualise the blood capillary network (CD31^+^LYVE‐1^‐^).^38^, ^39^ The effects of VEGF‐A inhibition in the epidermis were assessed using the proliferation marker Ki‐67 and measuring keratin 6 expression, which is upregulated in psoriatic epidermis.^40^ Lastly, we quantified the levels of mast cell activation, which contributes to psoriasis pathogenesis and constitutes an important source of VEGF‐A^20^, ^41^, ^42^; and key immune mediators in the pathogenesis of psoriasis, CD4^+^ and CD8^+^ cells. This showed reduced blood vessel area and EC numbers in plaques of psoriasis following VEGF‐A inhibition, which was especially pronounced in patients with high VEGF‐A levels in plasma and/or in organ culture supernatant, thus supporting our working hypothesis.

## 2 MATERIALS AND METHODS

### 2.1 Volunteers

Twelve individuals (6 with psoriasis and 6 without psoriasis) were recruited. Potential participants were excluded if they had used topical treatment on the biopsied area within 4 weeks or systemic treatments within 12 weeks of enrolment in the study; or had inflammatory arthropathy. The study was approved by the UK Health Research Authority (15/NW/0585, 09/H/101143) and adhered to the Declaration of Helsinki Guidelines. All subjects gave written, informed consent.

### 2.2 Skin sampling

Skin punch biopsies (3 mm) were collected from all donors. Those with psoriasis had two biopsies taken from plaques of psoriasis and two additional biopsies from non‐lesional skin (at least 5 cm away from plaques). Anatomical site‐to‐site variations were minimised by biopsy of the same body site (buttock). A detailed description of skin sampling is provided in Supplementary Files S1.

### 2.3 Human skin organ culture

Skin biopsies were incubated at an air‐liquid interface in 1 mL of supplemented EpiLife media (MEPI500CA, Gibco, ThermoFisher Scientific) for 72 h. To stimulate T cells and restore their inflammatory activity in skin organ culture, a T‐cell activation mix containing 1 μg/ml anti‐CD3 (clone ICHT1, cat: MAB100), 1 μg/ml anti‐CD28 (clone 37407, Cat: MAB342) and 50 ng/mL rh IL‐23 (Cat:1290‐IL; all from R&D Systems, Bio‐techne, Minnesota, USA) was added to the culture as previously described.^43^ A detailed description of skin organ culture and treatment is provided in Supplementary Files S1. Concentration of bevacizumab and duration of culture was iterated from our optimising study as previously described.^44^ Biopsies were incubated with 0.8 mg/mL of bevacizumab or 0.8 mg/mL of IgG_1_ isotype control (BioXCell, 2BScientific Ltd, Oxford, UK) for 72 h at 37°C and 5% CO_2_. Culture media was changed after 48 h. After 72 h, biopsies were embedded in optimal cutting temperature gel, snap frozen in liquid nitrogen and stored at −80°C until cryosectioning.

### 2.4 Histochemistry, immunofluorescence and quantitative immunohistomorphometry

Cryosections (8‐μm) were affixed to Superfrost Plus slides (Menzel‐Glaser, ThermoFisher Scientific). Immunofluorescence staining included: mouse anti‐CD31 (clone JC/70A, M0823, DAKO, 1:100); rabbit anti‐LYVE‐1 (ab10278, abcam, 1:100); rabbit anti‐Ki‐67 (ab16667, abcam, 1:50); mouse anti‐keratin 6 (ab18586, abcam, 1:200), mouse anti‐mast cell tryptase (ab2378, abcam, 1:500), mouse anti‐CD4 (clone 4B12, MA5‐12259, ThermoFisher, 1:50) and mouse anti‐CD8 (MA5‐144B, ThermoFisher, 1:100). DAPI was used to stain nuclei. Secondary antibodies used were goat anti‐rabbit 488 Alexa fluor (AF; 1:200), goat anti‐mouse 594 AF (1:200), goat anti‐mouse 488 Alexa fluor (1:200) and goat anti‐rabbit 594 AF (1:200). After staining, slides were mounted using Fluoromount mounting medium (S3023, Dako) and were visualised and photographed using a Keyence Biozero 8000 (Keyence Corporation, Osaka, Japan) or 3D Histec Pannoramic250 Slide scanner (Leica Biosystems, Wetzlar, Germany). Quantitative analysis was performed on QuPath v0.1.2 or ImageJ (Fiji). Morphometric analysis of blood vessel area, defined as the dermis area superficial to the deep vascular plexus (i.e., superficial vascular plexus and the capillary loop system) occupied by the blood vessels, was performed on ImageJ (Fiji) as previously described.^44^ A detailed description of immunofluorescence and quantitative histochemical analysis is provided in Supplementary Files S1.

### 2.5 Peripheral blood mononuclear cells culture

Venous blood was collected into EDTA‐tubes and peripheral blood mononuclear cells (PBMCs) were isolated using density gradient media – Lymphoprep ™ (STEMCELL Technologies Ltd, Cambridge, UK). Cells were incubated with lipopolysaccharide (LPS; L4391, Sigma‐Aldrich Inc, Munich, Germany) at 1 ng/mL or 5 ng/mL, as previously described.^45^ Cell culture supernatant were harvested and stored at −20°C before VEGF‐A assay. A detailed description of PBMC culture is provided in Supplementary Files S1.

### 2.6 Quantification of VEGF‐A levels

Levels of VEGF‐A protein in PBMCs culture supernatant and in organ culture supernatant were measured using an enzyme‐linked immunosorbent assay (ELISA; Duo set ELISA kit, R&D Systems Europe Ltd., Abingdon, UK). Plasma was separated from a portion of venous blood. A detailed description of plasma extraction is provided in Supplementary Files S1. Levels of VEGF‐A in plasma were measured using an ELISA (Quantikine ELISA, R&D Systems, Abingdon, UK) designed to assay both VEGF‐A_165_ and VEGF‐A_121_ isoforms. Optical density was determined using a microplate reader (CLARIOstar® plus, BMGLabtech).

### 2.7 DNA extraction and genotyping

Genomic DNA was isolated using QIAamp DNA blood midi kit (QIAGEN®, Crawley, UK). Genotyping of the −460 (rs833061) and +405 (rs2010963) VEGF‐A SNPs^15^, ^16^, ^37^ was performed using the SNP genotyping assay (Applied Biosystems, Foster City). A detailed description of DNA extraction and genotyping is provided in Supplementary Files S1.

### 2.8 Statistical analysis

Data analysis was performed using GraphPad Prism v8.1.2. Non‐normal distribution was assumed for continuous variables and results reported as median (interquartile range [IQR]), unless otherwise specified. The Wilcoxon matched pairs signed rank test was used to evaluate differences between control and treated samples for healthy, non‐lesional and plaque skin. Independent two‐sided t or Mann‐Whitney U tests were used to compare continuous variables between both groups. All tests were two‐sided and *p‐value < 0.05 was considered statistically significant. Non‐parametric Spearman's correlation was used to determine relationships between variables.

## 3 RESULTS

### 3.1 Inhibition of VEGF‐A with bevacizumab blocks free VEGF‐A in organ culture

Skin samples were taken from 5 women and 1 man with psoriasis, with median age of 46.5 years (inter‐quartile range ([IQR] 27.75), median age of psoriasis onset 24 years (IQR 17) and median PASI (psoriasis area and severity index) of 12.65 (IQR 9.8). 50% (*n* = 3) had a family history of psoriasis. Skin samples were taken from 6 healthy controls with median age of 42.5 years (IQR 14.5; Table 1). Psoriasis plaque skin maintained its psoriatic phenotype ex vivo, including increased rete ridge length, epidermal area, epidermal hyperproliferation and keratin 6 expression compared to non‐lesional and healthy skin (Figure S1).

**TABLE 1:** Demographic details and clinical characteristics of donors.

Patients with psoriasis							
Volunteer ID	PS1	PS2	PS3	PS4	PS5	PS6	Median (IQR)
Sex	F	F	F	F	F	M	
Age (years)	42	51	57	61	28	31	46.5 (27.75)
Age of onset	11	24	24	58	19	26	24 (17)
Disease duration	31	28	33	3	9	5	18.5 (27)
Family history	Yes	Yes	No	No	No	Yes	
Severity	10/10	8/10	3/10	3/10	5/10	5/10	5 (5.5)
PASI	16	21.8	6.6	8.5	9.3	16.5	12.65 (9.8)
PEST	4/5	2/5	0/5	0/5	3/5	2/5	2 (3.25)
DLQI	25/30	5/30	10/30	14/30	13/30	25/30	13.5 (16.25)
Finger nails	Yes (3)	No	No	No	Yes (3)	Yes (10)	1.5 (4.75)
Toe nails	Yes (3)	Yes (10)	No	No	Yes (2)	Yes (1)	1.5 (4.75)
Height (m)	1.72	1.67	1.69	1.55	1.72	1.88	1.7 (0.12)
Weight (kg)	101.4	119	85.4	78.4	111.1	136.2	106.3 (39.65)
BMI	34.2	42.6	29.9	32.6	37.5	38.5	35.91 (7.62)
SBP (mm Hg)	116	129	177	131	136	128	130.5 (21.3)
DBP (mm Hg)	77	84	107	82	80	84	83 (10.5)
VEGF‐A polymorphism							
+405	G/G	C/G	C/C	C/G	G/G	C/G	
−460	C/C	C/T	T/T	T/T	C/C	C/T	
VEGF‐A plasma level (pg/ml)	67.2	27.6	13	23	16.4	81.4	25.3 (55.21)

Healthy volunteers							
Volunteer ID	H1	H2	H3	H4	H5	H6	Median (IQR)
Sex	F	F	M	M	F	F	
Age	38	49	46	39	54	29	42.5 (14.5)
Height (m)	1.6	1.6	1.82	1.8	1.6	1.6	1.68 (0.18)
Weight (kg)	102.4	92	121.8	96.3	66.6	65	96.3 (32.8)
BMI	39	33.8	36.7	29.4	23.6	23.3	33.79 (11.39)
SBP (mm Hg)	110	138	126	124	98	119	124 (22)
DBP (mm Hg)	80	78	100	86	68	77	80 (14.75)
VEGF‐A polymorphism							
+405	G/G	N/A	C/C	G/G	G/G	G/G	
−460	C/T	N/A	T/T	C/C	C/C	C/T	
VEGF‐A plasma level (pg/ml)	2.6	38.5	71	3	14.7	13.5	14.13 (43.65)

*Note*: Values are expressed as median (range).

Abbreviations: BMI, body mass index; DBP, diastolic blood pressure; DLQI, Dermatology Life Quality Index; H, healthy; IQR, interquartile range (25^th^‐75^th^ percentiles); N/A, Not available; PASI, Psoriasis Area Severity Index; PEST, Psoriasis Epidemiology Screening Tool; PS, Psoriasis; SBP, systolic blood pressure.

**FIGURE 1:**
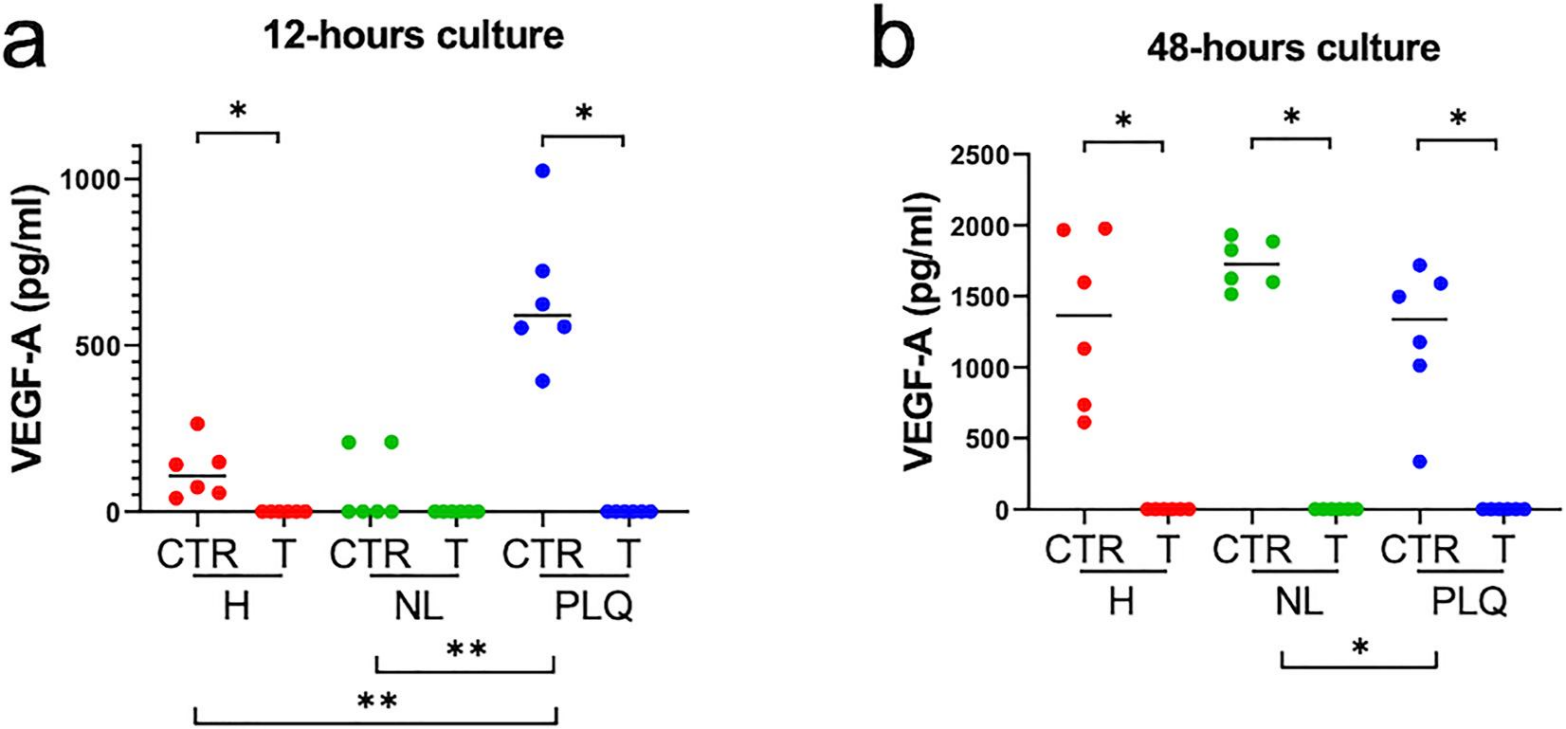
Bevacizumab blocks free VEGF‐A in organ culture. VEGF‐A levels were measured in culture supernatant of organ‐cultured healthy, non‐lesional and plaque skin after 12 and 48 h using an ELISA. VEGF‐A was undetectable in organ culture supernatant of healthy, non‐lesional and psoriasis plaque skin 12 and 48 h after incubation with bevacizumab. (a) At 12 h, the levels of VEGF‐A in organ culture supernatant of isotype control‐treated plaque were higher than those of isotype control‐treated non‐lesional (**p < 0.01) and isotype control‐treated healthy skin (**p < 0.01). (b) At 48 h, the levels of VEGF‐A in organ culture supernatant of isotype control‐treated non‐lesional skin (1726 [320]) were higher than those of isotype control‐treated plaque (1338 [779.3]; *p < 0.05). Number of independent experiments *n* = 36 (1 punch biopsy per patient and per treatment group). Data were presented as median and were analysed with two‐tailed Wilcoxon matched‐pairs signed rank test (paired analysis) or two‐tailed Mann Whitney test (unpaired analysis). CTR, isotype control; H, healthy; NL, non‐lesional; PLQ, plaque; T, treated with anti‐VEGF‐A monoclonal antibody.

First, we investigated whether bevacizumab effectively blocked VEGF‐A, showing that VEGF‐A was undetectable at 12 and 48 h in culture supernatant of healthy, non‐lesional and plaque skin incubated with 0.8 mg/mL of bevacizumab (Figure 1a,b). At 12 h, there were significantly higher levels of VEGF‐A in culture media of isotype‐control treated plaque compared to isotype control‐treated non‐lesional (**p < 0.01) and healthy skin (**p < 0.01, Figure 1a). Therefore, bevacizumab effectively blocks free VEGF‐A in human skin organ culture.

### 3.2 VEGF‐A inhibition downregulates angiogenesis in plaques of psoriasis

**FIGURE 2:**
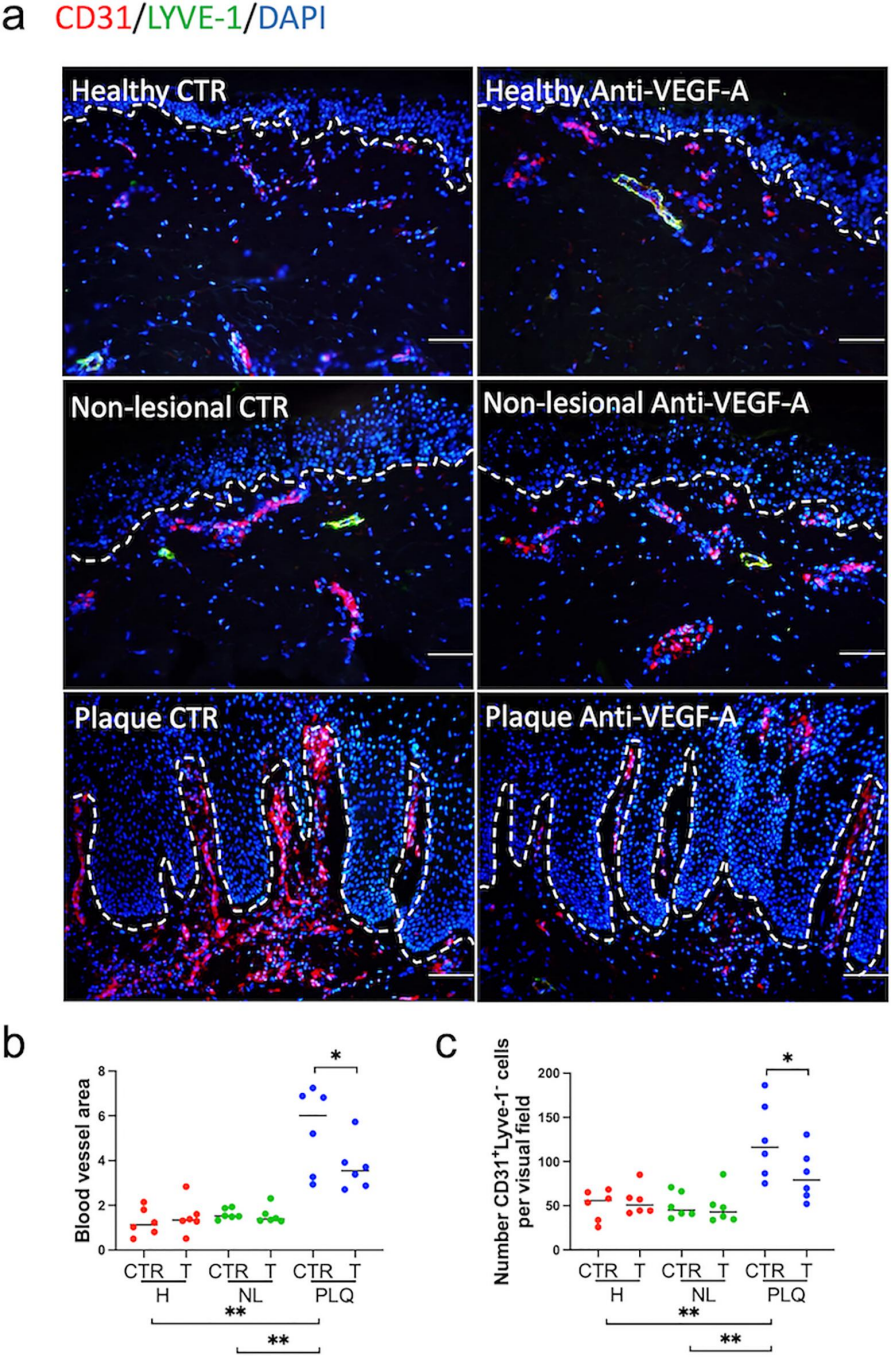
VEGF‐A blockade decreased blood vessel area and the number of blood endothelial cells in psoriasis plaque skin (a) Blood vessel area was assessed using double immunofluorescence stain CD31 (red)/LYVE‐1 (green). DAPI stained the nuclei. (b) Blood vessel area was higher in isotype control‐treated plaque (6 [3.78]) compared to isotype control‐treated non‐lesional (1.52 [0.43]; **p < 0.01) and isotype control‐treated healthy (1.13 [1.15]; **p < 0.01). (c) The number of blood endothelial cells (ECs) was higher in isotype control‐treated plaque (116.2 [84.45]) compared to isotype control‐treated non‐lesional (44.97 [27.9]; **p < 0.01) and isotype control‐treated healthy (55.8 [34.34]; *p* < 0.01). Bevacizumab decreased (a) blood vessel area (3.54 [1.53]; *p* < 0.05) and (b) the number of CD31^+^LYVE‐1^‐^ ECs (79.1 [50.72]; *p < 0.05) in plaques of psoriasis. Number of independent experiments *n* = 36 (1 punch biopsy per patient and per treatment group). Data were presented as median and were analysed with two‐tailed Wilcoxon matched‐pairs signed rank test (paired analysis) or two‐tailed Mann Whitney test (unpaired analysis). Scale bars = 100 μm. *p < 0.05, **p < 0.01. CTR, isotype control; H, healthy; NL, non‐lesional; PLQ, plaque; T, treated with anti‐VEGF‐A monoclonal antibody.

Next, we asked whether VEGF‐A inhibition downregulates angiogenesis in skin organ culture using CD31/LYVE‐1 staining (Figure 2a). Blood vessel area, defined as the dermis area distal to the deep vascular plexus occupied by CD31^+^LYVE‐1^‐^ blood vessels, was significantly higher in isotype control‐treated plaque (6 [3.78]) compared to isotype control‐treated non‐lesional (1.52 [0.43]; **p < 0.01) and isotype control‐treated healthy skin (1.13 [1.15]; **p < 0.01; Figure 2b). After 3 days of anti‐VEGF‐A treatment ex vivo, the blood vessel area in bevacizumab‐treated, organ‐cultured plaque was significantly lower (3.54 [1.53]) than in isotype control‐treated plaque (6 [3.78]; *p < 0.05; Figure 2b).

As expected from the literature,^46^, ^47^, ^48^ the number of CD31^+^LYVE‐1^‐^ ECs in the dermis distal to the deep vascular plexus was significantly higher in isotype control‐treated plaque (116.2 [84.45]) compared to isotype control‐treated non‐lesional (44.97 [27.9]; **p < 0.01) and isotype control‐treated healthy skin (55.8 [34.34]; **p < 0.01; Figure 2c). After bevacizumab treatment, the number of ECs in plaques declined significantly (79.1 [50.72]), below that of isotype control‐treated plaques (116.2 [84.45]; *p < 0.05; Figure 2c). These data demonstrate that short‐term anti‐VEGF‐A therapy downregulates angiogenesis in plaques of psoriasis ex vivo.

### 3.3 VEGF‐A inhibition does not significantly change epidermal read‐out parameters

**FIGURE 3:**
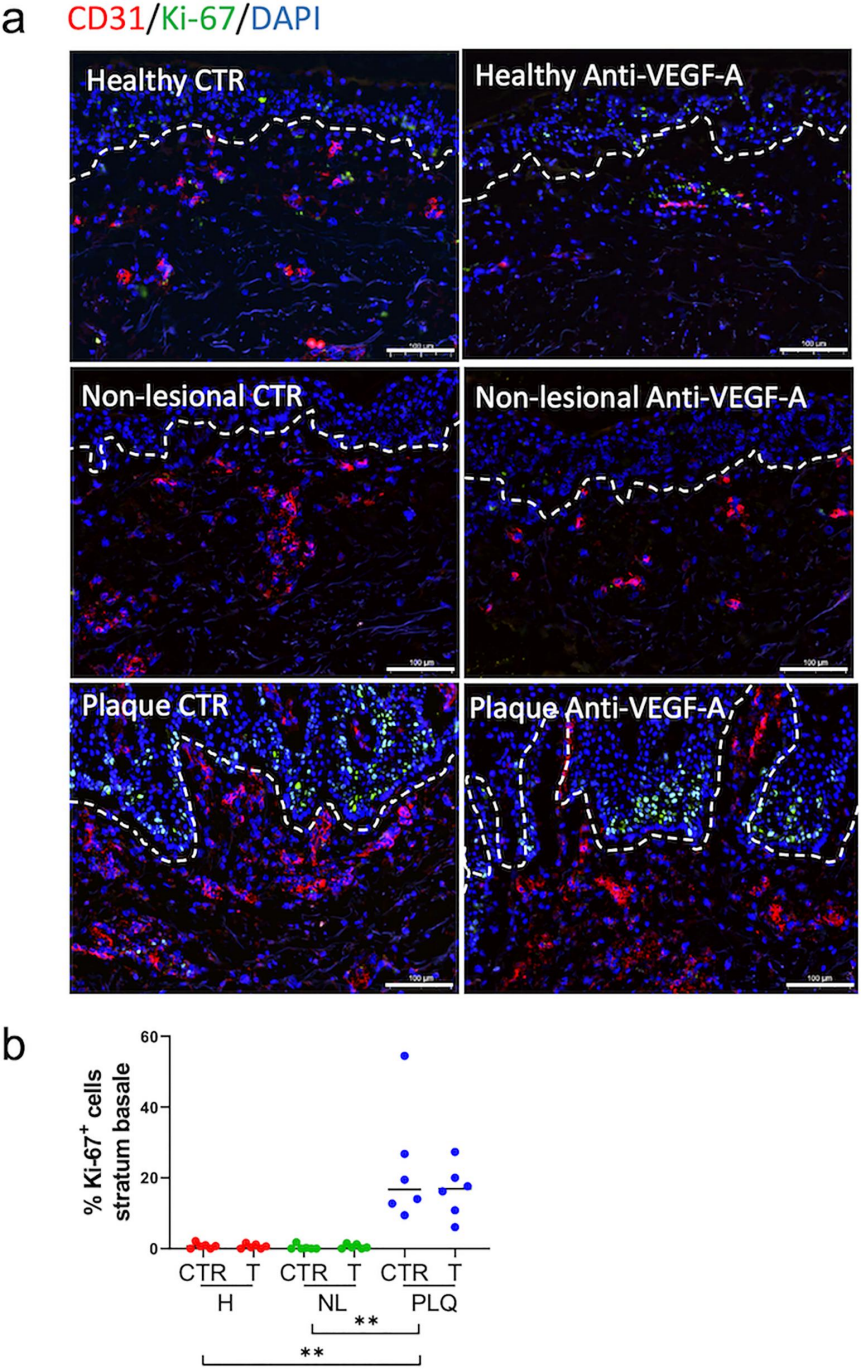
VEGF‐A inhibition does not affect epidermal proliferation in psoriasis plaque skin ex vivo (a) Proliferation was assessed using double immunofluorescence staining CD31 (red)/Ki‐67 (green) in the stratum basale (SB). DAPI stained the nuclei. (b) Proliferation in the SB was higher in isotype control‐treated plaques (16.74 [21.79]) than in isotype control‐treated non‐lesional (0 [0.61); **p < 0.01) and isotype control‐treated healthy skin (0.37 [1.76]; **p < 0.01). Number of independent experiments *n* = 36 (1 punch biopsy per patient and per treatment group). Data were presented as median and were analysed with two‐tailed Wilcoxon matched‐pairs signed rank test (paired analysis) or two‐tailed Mann Whitney test (unpaired analysis). Scale bars = 100 μm. **p < 0.01. CTR, isotype control; H, healthy; NL, non‐lesional; PLQ, plaque; T, treated with anti‐VEGF‐A monoclonal antibody.

We assessed the effects of bevacizumab on the epidermis, finding that VEGF‐A inhibition did not affect rete ridge length, epidermal length, epidermal area or keratin 6 expression in plaques ex vivo (Figure S1). After 3 days organ culture, the number of proliferating keratinocytes in the stratum basale (SB) was significantly higher in isotype control‐treated plaque (16.74 [21.79]) compared to isotype control‐treated non‐lesional (0 [0.61]; **p < 0.01) and isotype control‐treated healthy skin (0.68 [1.25]; **p < 0.01; Figure 3a,b). VEGF‐A inhibition did not affect TUNEL expression in the epidermal SB (Figure S1). This suggests that short‐term anti‐VEGF‐A therapy hardly impacts on key epidermal parameters, at least ex vivo.

### 3.4 VEGF‐A inhibition does not significantly change the number of tryptase^+^‐mast cells, CD4^+^ or CD8^+^ T‐cells in plaques of psoriasis

We also investigated ex vivo the impact of VEGF‐A inhibition on T‐cell localisation and dermal mast cells (MCs). The number of tryptase^+^‐MCs (mast cell tryptase^+^ DAPI^+^) was significantly higher in isotype control‐treated plaque (15.92 [15.47]) compared to isotype control‐treated non‐lesional (7.81 [9.9]; *p < 0.05) and isotype control‐treated healthy skin (5.67 [7.01]; *p < 0.05; Figure S2.

The number of CD4^+^ T‐cells was significantly higher in the dermis (33.71 [20.9]) and epidermis (1.67 [2.85]) of isotype control‐treated plaque compared to the dermis (8.43 [0.86]; **p < 0.01) and epidermis (0; **p < 0.01) of isotype control‐treated non‐lesional skin, respectively. The number of CD8^+^ T‐cells was significantly higher in the dermis of isotype control‐treated plaque (19.28 [9.86]) compared to the dermis of isotype control‐treated non‐lesional skin (3.66 [3.73]; **p < 0.01; Figures S3 and S4). Although the number of tryptase^+^‐MCs increased in bevacizumab‐treated plaque (24.81 [25.37]) compared to isotype control‐treated plaque (15.92 [15.47]), this did not reach significance (*p* = 0.15, Figure S2). VEGF‐A inhibition also did not significantly alter the number of CD4^+^ or CD8^+^ cells in the dermis or epidermis (Figure S3 and S4).

### 3.5 Anti‐angiogenic response to bevacizumab correlates with levels of VEGF‐A in the plasma and plaque skin

Finally, we asked whether differences in the anti‐angiogenic response to VEGF‐A inhibition were determined by levels of VEGF‐A in plasma or skin, disease severity, VEGF‐A polymorphisms or PBMC production of VEGF‐A. There were no differences in VEGF‐A genotype frequencies at the +405 or −460 sites between our donors when comparing healthy volunteers with patients with psoriasis or between volunteers with severe psoriasis and volunteers with mild to moderate psoriasis (Tables S1 and S2). Thus, no association between genotype and response to anti‐VEGF‐A treatment could be identified.

**FIGURE 4:**
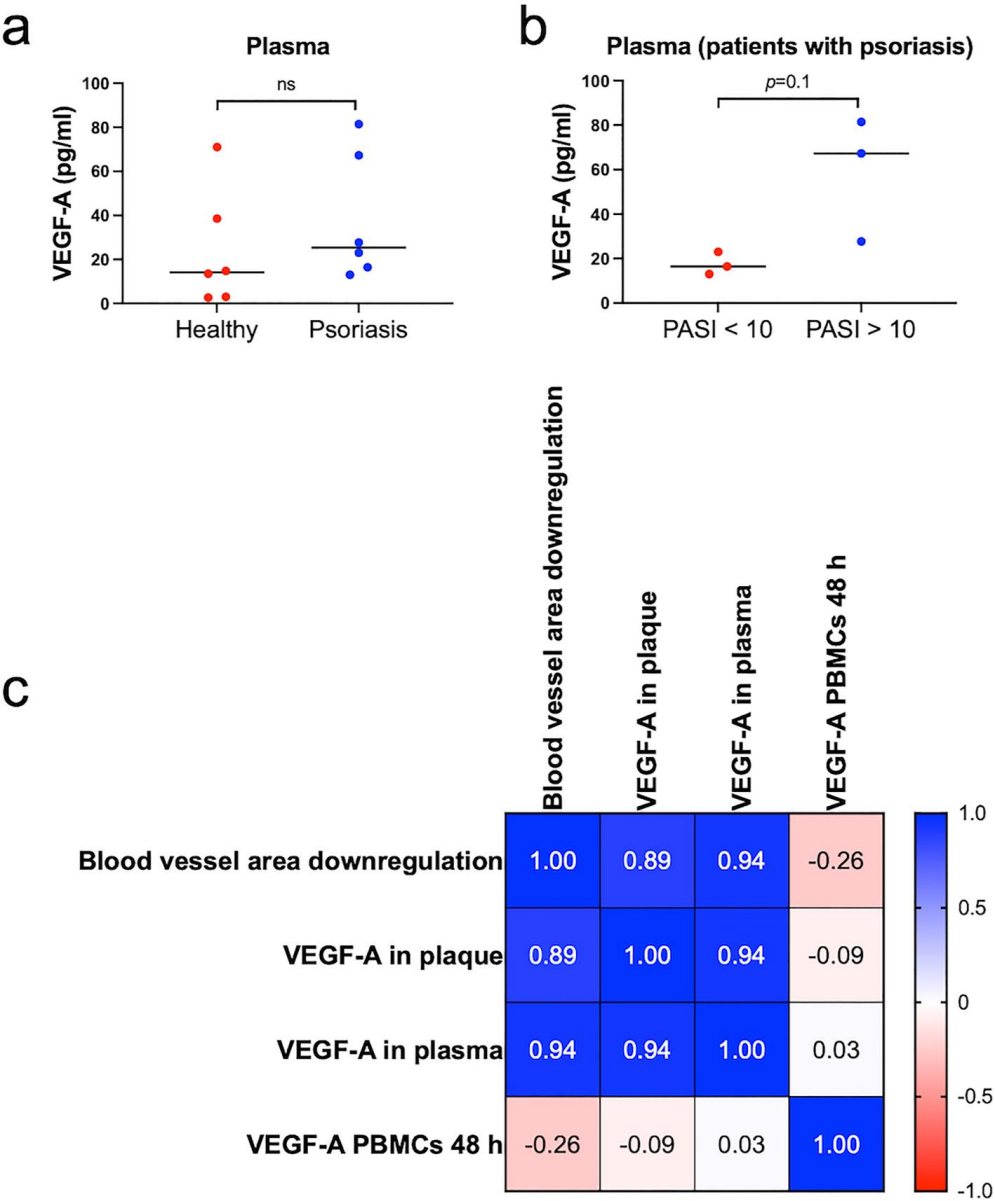
Blood vessel area downregulation correlated with the levels of VEGF‐A in plasma and VEGF‐A production in psoriasis plaque skin (a) The median level of VEGF‐A in plasma of patients with psoriasis and healthy volunteers was 25.38 (range 13–81) pg/ml and 14.13 (3–71) pg/ml, respectively. (b) The levels of VEGF‐A in plasma in volunteers with psoriasis with PASI<10 were not significantly different to those with PASI>10 (*p* = 0.10). (c) There was a positive correlation between blood vessel area downregulation in plaque skin and VEGF‐A levels in culture supernatant of plaque skin at 12 h (*r* = 0.89, *p* < 0.05). Blood vessel area downregulation also correlated positively with VEGF‐A levels in plasma of patients with psoriasis (*r* = 0.94; *p* < 0.05). There was a positive correlation between VEGF‐A levels in culture supernatant of plaque skin at 12 h and VEGF‐A levels in plasma of patients with psoriasis (*r* = 0.94; *p* < 0.05). Data were analysed using two‐tailed Mann‐Whitney test. Correlations were analysed using Spearman correlation coefficient (*r*). PBMCs, peripheral blood mononuclear cells.

The VEGF‐A plasma levels between all patients with psoriasis and healthy volunteers, overall, showed no significant differences (Figure 4a). However, patients with severe disease (PASI>10: *n* = 3), tended towards exhibiting higher VEGF‐A plasma levels than patients with moderate disease (PASI<10; *n* = 3), although this did not reach significance (*p* = 0.1; Figure 4b). VEGF‐A production by PBMCs did not differ between healthy volunteers and psoriasis patients (Figure S5), and VEGF‐A production by PBMCs did not correlate with response to anti‐VEGF‐A treatment (Figure 4c). VEGF‐A plasma levels of patients with psoriasis correlated well with VEGF‐A levels measured in culture supernatant of plaques at 12 h (*r* = 0.94, *p < 0.05; Figure 4c).

Importantly, we noted a positive correlation between bevacizumab‐induced blood vessel area downregulation in 3‐day organ‐cultured psoriasis plaques ex vivo with the VEGF‐A plasma levels of psoriasis patients (*r* = 0.94, *p < 0.05; Figure 4c). Bevacizumab‐induced blood vessel area downregulation in plaque also correlated with the levels of VEGF‐A in culture supernatant of isotype control‐treated plaque at 12 h (*r* = 0.89, *p < 0.05; Figure 4c). Despite the relatively low numbers that could be examined, these ex vivo/in vivo correlations suggest that the anti‐angiogenic response to bevacizumab is greater in those with higher levels of VEGF‐A in the skin and/or plasma.

## 4 DISCUSSION

In this ex vivo pilot study, we demonstrate that VEGF‐A inhibition by bevacizumab exerts significant anti‐angiogenic activity in plaques of psoriasis. In agreement with previous studies,^16^, ^49^ patients with severe psoriasis (PASI >10) had higher levels of VEGF‐A in plasma and plaques of psoriasis. Despite the limited number of skin biopsies and donors that were available for study, our preclinical data encourage one to systematically explore clinically whether blocking VEGF‐A is an effective adjunct management strategy for downregulating psoriasis‐associated pathological angiogenesis. We therefore hypothesise that this treatment strategy may be most effective in patients with high levels of VEGF‐A in the skin and/or plasma.

The ability of VEGF‐A inhibition to downregulate pathological angiogenesis has been demonstrated in pre‐clinical studies of cancer and is well established.^50^, ^51^, ^52^, ^53^, ^54^, ^55^ Bevacizumab, which is currently used to downregulate angiogenesis in the treatment of metastatic colorectal cancer,^54^, ^55^, ^56^, ^57^ non‐squamous non‐small cell lung cancer^58^, ^59^ and hepatic cell carcinoma,^60^ is used as a long‐term treatment and is often combined with chemotherapeutic agents.^61^, ^62^, ^63^ To the best of our knowledge, we describe here the first studies to explore a potential therapeutic effect in inflammatory skin disease, demonstrating that VEGF‐A inhibition by bevacizumab exerts significant anti‐angiogenic activity in skin organ culture of psoriasis. Manifest by significant diminution in blood vessel area and blood vessel ECs in plaques of psoriasis, response to bevacizumab was significantly greater in patients with high levels of VEGF‐A in plasma/plaque skin and appeared to offer most treatment benefit to those with severe psoriasis. These preclinical data encourage further exploration of whether downregulation of pathological angiogenesis represents an adjuvant management strategy for patients with psoriasis. Although we observed no histological resolution of psoriasis, this was expected, given the short incubation time (72 h). However, early knockdown of the blood vasculature and prolonged VEGF‐A inhibition could induce downregulation of epidermal hyperplasia due to keratinocyte starvation. While further research is required to test this hypothesis, psoriasis management could be optimised in the future through downregulation of angiogenesis in conjunction with the simultaneous inhibition of key regulatory pathways in psoriasis such as IL‐17/IL‐23.

Long‐term inhibition of VEGF‐A may lead to undesired age‐promoting effects^64^, ^65^ and, therefore, anti‐VEGF‐A therapy for the management of psoriasis may best be administered as repetitive pulse therapy. In addition, nanotechnology‐based angiogenesis‐targeting strategies could be used to minimise the potential systemic complications associated with tissue ageing effects of blocking VEGF‐A, as these can also lead to renal and cardiovascular impairment.^66^, ^67^ However, anti‐VEGF‐A therapy could be beneficial for patients with psoriasis through a direct effect on both the disease and its systemic sequelae,^68^, ^69^, ^70^ perhaps reducing the risk of psoriasis‐associated vascular events (myocardial infarction and stroke), and cardio‐metabolic diseases (hypertension, diabetes mellitus and metabolic syndrome).^71^, ^72^

We also show that VEGF‐A inhibition increases the number of tryptase^+^‐MCs in plaques. Since our skin organ cultures had no access to circulating MC progenitors, this could reflect a promoting effect on the differentiation of mature MCs from resident mast cell progenitor cells that we have previously shown to be present in human skin mesenchyme under organ culture conditions.^73^, ^74^ Tryptase, a major secretory product of human MCs, is a marker of MC activation^75^ and a mitogen for dermal ECs,^76^, ^77^ and the release of pro‐inflammatory and pro‐angiogenic mediators from MCs can decrease the efficacy of anti‐angiogenic therapy in tumours.^78^, ^79^ Therefore, it deserves further exploration whether the efficacy of an anti‐angiogenic psoriasis management strategy can be further enhanced by combining it with inhibitors of MC degranulation.

A recent prospective cohort study identified a correlation between the degree of nail fold capillary (NFC) changes and the severity of psoriatic arthritis (PsA), suggesting that NFC abnormalities may be an indicator of PsA progression.^80^ Anti‐VEGF therapy may not only be effective against psoriasis skin lesions, but may also inhibit the progression to PsA.

The study had limitations. First, a limited number of biopsies and donors were available and further investigations in a larger group of patients are required to identify associations between VEGF‐A genetic signatures and response to treatment. However, our experimental protocol was carefully and uniformly performed from various viewpoints, thus informing future investigations. Second, the ex vivo nature of the study and lack of tissue perfusion prevented investigation of histological changes over a prolonged period of time. Nevertheless, skin organ culture allows instructive study the effects of VEGF‐A inhibition on the vasculature, including all skin compartments and resident skin cells. Finally, skin organ culture may be intrinsically affected by a wound healing response at 48 h, and we observed levels of VEGF‐A increasing, in all groups in keeping with this.^81^ However, despite this, bevacizumab effectively blocked all free VEGF‐A in culture supernatant of healthy, non‐lesional and plaque skin ex vivo after 72 h.

In conclusion, this pilot study provides proof‐of‐principle for the investigation of VEGF‐A inhibition as an adjuvant management strategy to selectively target vascular pathology in psoriasis. This approach could be especially beneficial for patients who have high levels of VEGF‐A, offering an opportunity to personalise management and complement current anti‐cytokine strategies and other standard‐of‐care psoriasis therapeutics.

## CONFLICT OF INTEREST STATEMENT

The authors state no conflict of interest.

## AUTHOR CONTRIBUTIONS

**Andrea Luengas‐Martinez**: conceptualization (supporting); data curation (equal); formal analysis (equal); investigation (equal); methodology (equal); validation (equal); writing – review & editing (lead); writing – review & editing (lead). **Dina Ismail**: data curation (supporting); investigation (equal); methodology (supporting); resources (supporting); writing – original draft (supporting); writing – review & editing (supporting). **Ralf Paus**: conceptualization (supporting); data curation (supporting); formal analysis (supporting); funding acquisition (supporting); investigation (supporting); methodology (supporting); resources (supporting); supervision (supporting); writing – original draft (supporting); writing – review & editing (supporting). **Helen S. Young**: conceptualization (lead); data curation (supporting); formal analysis (supporting); funding acquisition (lead); investigation (supporting); methodology (lead); project administration (lead); resources (lead); supervision (lead); visualization (lead); writing – original draft (supporting); writing – review & editing (lead).

## ETHICS STATEMENT

The study was approved by the UK Health Research Authority (15/NW/0585, 09/H/101143) and adhered to the Declaration of Helsinki Guidelines. All subjects gave written, informed consent.

## Supporting information

Supporting Information S1

Supporting Information S2

## Data Availability

No datasets were generated or analysed during the current study.
