# Supplementary material for: Inhibition of vascular endothelial growth factor‐A downregulates angiogenesis in psoriasis: A pilot study
Source: Skin Health Dis. 2023 May 15;3(5):e245. doi: 10.1002/ski2.245 (PMC10549813; doi:10.1002/ski2.245)
Supplement: Supplementary file 1 — Supporting Information S1 [file SKI2-3-e245-s001.docx]

**SUPPLEMENTARY FILES**

**Supplementary Materials and Methods**

**1 Skin sampling**

Skin punch biopsies (3 mm) were collected from the buttocks of healthy and volunteers with psoriasis and placed in culture within maximum 3 hours after removal. Prior to taking the biopsy, the skin was sterilised with chlorhexidine and local anaesthesia was achieved with an injection of lidocaine hydrochloride (1 % 50 mg/5 ml, PHA075, Hameln pharmaceuticals, Gloucester, UK) without adrenaline to preserve the blood vessels integrity. For patients with psoriasis, two biopsies were taken from plaque skin (within the centre of the plaque) and two biopsies were taken from non-lesional skin (at least 5 cm away from any plaque). Biopsies were transported over to the laboratory from Salford Royal in William’s E medium (Gibco™, Thermo Fisher Scientific, Massachusetts, USA) supplemented with 100 U/ml penicillin, 10 mg/mL Streptomycin (Gibco™, Thermo Fisher Scientific) and 2 mM L-glutamine (Invitrogen™, Thermo Fisher Scientific).

**2 Human skin organ culture**

Skin biopsies were incubated at an air-liquid interface in 1 ml of supplemented EpiLife™ media (MEPI500CA, Gibco™, Thermo Fisher Scientific) containing 60 µm calcium (MEPI500CA, Gibco™, Thermo Fisher Scientific). The supplements included: 0.2 ng/ml human epidermal growth factor, 0.2 % bovine pituitary extract, 5 µg/ml bovine insulin, 5 µg/ml bovine transferrin, 250 ng/ml Amphotericin B and 10 µg/ml Gentamicin (all from human keratinocyte growth supplement kit, S-001-K, Gibco™, Thermo Fisher Scientific), 10 ng/ml recombinant human (rh) IL-2, 100 U/ml penicillin and 100 µg/ml streptomycin. For *in situ* activation of T cells, a T cell activation mix containing 1 µg/ml anti-CD3 (clone ICHT1, cat: MAB100), 1 µg/ml anti-CD28 (clone 37407, Cat: MAB342) and 50 ng/ml rh IL-23 (Cat:1290-IL; all from R&D Systems, Bio-techne, Minnesota, USA) was used. ^1^ For culture at an air-liquid interface, ThinCert® Cell Culture inserts (Greiner Bio-One, Gloucestershire, UK) were pierced in the centre and the biopsy was inserted in the centre.

**3 Treatment of organ culture**

Skin biopsies were incubated overnight in 1 ml of supplemented EpiLife™ media to adapt to culture conditions. After, the media was replaced by media containing T cell activation mix and either 0.8 mg/ml of bevacizumab (Avastin®, Roche, Basel, Switzerland) or 0.8 mg/ml of IgG_1_ isotype control (BioXCell, 2BScientific Ltd, Oxford, UK) for 72 hours at 37 °C and 5 % CO_2_. The dose of bevacizumab was iterated from our pilot study in healthy human skin. In this study we tested three different concentrations of bevacizumab (0.01, 0.2 and 0.8 mg/ml). ^2^ The highest concentration (0.8 mg/ml) neutralised all VEGF-A present in organ culture supernatant after at 72 hours and inhibited VEGF-A mediated endothelial cell survival in healthy human skin *ex vivo.* ^2^ Culture media was changed once after 48 hours. After 72 hours, biopsies were embedded in OCT, snap frozen in liquid nitrogen and stored at - 80 °C until cryosectioning.

**4 Histochemistry and immunofluorescence staining**

The following primary antibodies and dilutions were used for immunofluorescence staining of cryosections of healthy and psoriasis skin: mouse anti-CD31 (clone JC/70A, M0823, DAKO, 1:100); rabbit anti-Lyve-1 (ab10278, abcam, 1:100); rabbit anti-Ki-67 (ab16667, abcam, 1:50); mouse anti-keratin 6 (ab18586, abcam, 1:200), mouse anti-mast cell tryptase (ab2378, abcam, 1:500), mouse anti-CD4 (clone 4B12, MA5-12259, thermo fisher, 1:50) and mouse anti-CD8 (MA5-144B, thermo fisher, 1:100). DAPI was used to stain the nuclei. The secondary antibodies used were goat anti-rabbit 488 alexa fluor (AF; 1:200), goat anti-mouse 594 AF (1:200), goat anti-mouse 488 alexa fluor (1:200) and goat anti-rabbit 594 AF (1:200). The terminal deoxynucleotidyl transferase (TdT) dUTP Nick-End Labeling (TUNEL) assay (ApopTag® Plus Fluorescein, Millipore Corporation, Massachusetts, USA) was used for the detection of cell death. Anti-mouse IgG, DyLight® 488 (VectaFluor™ Excel Amplified Kit, Vector laboratories, Inc, Burlingame, CA) was used for detection of CD4 and CD8 antibodies. After staining, slides were mounted using Fluoromount mounting medium (S3023, Dako) and were visualised and photographed using a Keyence Biozero 8000 (Keyence Corporation, Osaka, Japan) or 3D Histec Pannoramic250 Slide scanner (Leica Biosystems, Wetzlar, Germany). Detailed description of immunofluorescence protocols has been described before. ^2^

**5 Quantitative histochemical analysis**

Haematoxylin and eosin (H&E) images were used to measure rete ridge length (at the longest rete ridge). The rete ridge length was defined as the vertical distance from the bottom of the stratum basale to the cusp of the rete ridge in the epidermis layer. Rete ridges were assessed in 4 sections per volunteer and per condition and a minimum number of 10 rete ridges per section were measured and the average was calculated. Epidermis area (µm^2^) was defined as the area from the stratum basale to the stratum granulosum, measured in 1 mm length of epidermis. Epithelial length (µm) was defined as the distance from one end of the biopsy to the other end.

**6 Quantitative Immunofluorescence analysis**

*Assessment of blood vessel area in the dermis superficial to the deep vascular plexus*

Sections stained for CD31/LYVE-1/DAPI were used to quantify blood vessel area. Blood vessel area was defined as the dermis area superficial to the deep vascular plexus (i.e., superficial vascular plexus and the capillary loop system) occupied by the blood vessels. For each skin biopsy, blood vessel area (CD31^+^/LYVE-1^-^/DAPI^+^) was measured in a minimum of 8 microscopic fields of view at 200X magnification and the average was calculated. Areas unsuitable for analysis such as tissue folds, areas surrounding hair follicles, the biopsy edges and other artefacts were excluded from the analysis. A macro for ImageJ (Fiji), which identifies and segments blood vessels automatically, was used for the computer-assisted morphometric analysis of blood vessels as in previous studies. ^2^ The macro can be found in the following repository:

<https://github.com/gadeamm/MacroAnalysisBloodVessels>. Images were visualised and photographed using a Keyence Biozero 8000 (Keyence Corporation).

*Quantification of blood endothelial cells in dermis superficial to the deep vascular plexus*

Sections stained for CD31/LYVE-1/DAPI were used to count the number of blood endothelial cell nuclei. For each patient, the number of blood endothelial cell nuclei (CD31^+^LYVE-1^-^DAPI^+^) were counted in the dermis superficial to the deep vascular plexus (superficial vascular plexus and in capillary loop system) in a minimum of 8 microscopic fields of view at 200X magnification and expressed as an average number of endothelial cells per field of view.

*Quantification of keratinocyte proliferation in the stratum basale*

Sections stained for CD31/Ki-67/DAPI were used to assess keratinocyte proliferation in the stratum basale. For each skin biopsy, the number of proliferating keratinocytes (Ki-67^+^ DAPI^+^) was quantified across the entire epithelial length of at least three non-consecutive sections and was expressed as the percentage of Ki-67^+^ cells in the stratum basale. Images were acquired on a 3D-Histech Pannoramic-250 microscope slide-scanner using a 20X/ 0.8 Plan Apochromat objective (Zeiss) and the DAPI/ FITC and TRITC filter sets. Quantitative analysis was performed on QuPath (v0.1.2). ^3^

*Mast cell tryptase^+^ cells quantification*

Sections stained for mast cell tryptase/DAPI were used to assess the number of tryptase^+^ mast cells in the dermis. For each skin biopsy, the number of cells expressing tryptase in the cytoplasm (mast cell tryptase^+^DAPI^+^) was quantified in at least 6 areas of 900 µm^2^ in the dermis area superficial to the deep vascular plexus in at least three different non-consecutive sections. The number of tryptase^+^ mast cells was expressed as mast cells tryptase^+^ cells. Images were acquired on a 3D-Histech Pannoramic-250 microscope slide-scanner using a 20X/ 0.8 Plan Apochromat objective (Zeiss) and the DAPI/ FITC and TRITC filter sets. Quantitative analysis was performed on QuPath v0.1.2. ^3^

*CD4^+^ and CD8^+^ cells quantification*

Sections stained for CD4/DAPI and CD8/DAPI were used to assess the number of CD4^+^ T cells and CD8^+^ cells, respectively. For each skin biopsy, the number of cells expressing CD4 (CD4^+^DAPI^+^) and CD8 (CD8^+^DAPI^+^) was quantified in at least 5 areas of 900 µm^2^ in the dermis area superficial to the deep vascular plexus in at least three different non-consecutive sections. In the epidermis, the number of CD4^+^DAPI^+^ and CD8^+^DAPI^+^  were counted on 3 randomly selected areas of the epidermis with a width of 200 µm^2^. For each skin biopsy, at least 5 areas from at least three different non-consecutive sections were quantified.

*Quantification of keratin 6 expression*

Sections stained for keratin 6/DAPI were used to assess keratin 6 expression in the epidermis. For each skin biopsy, the mean fluorescent intensity of keratin 6 was quantified in the epidermis of at least 10 fields of view using ImageJ (Fiji).^4^ Images were visualised and photographed using a Keyence Biozero 8000 (Keyence Corporation).

**7 Plasma extraction**

10 ml of blood were used immediately for plasma extraction. Blood was centrifuged at 2000 x g for 10 minutes at room temperature and was transferred to a new tube avoiding the blood pellet at the bottom. The centrifugation was repeated at the same settings, the double-spun plasma was transferred to a new tube avoiding any cell pellet that had formed at the bottom and the plasma was mixed by pipetting up and down. 1 ml plasma aliquots were prepared in Sarstedt Cryovials and samples were frozen immediately and stored at -80 °C.

**8 Peripheral blood mononuclear cells culture**

A cell culture model was established to provide a system to study VEGF-A production in PBMCs given a specific stimulus and under standardised, controlled conditions. Viable PBMCs were counted using Trypan blue 0.4 % solution (17-942E, Lonza). PBMCs were cultured at a fixed concentration of 1x10^6^ cells/ml in a humidified incubator at 37 °C and 5 % CO_2._. PBMCs were resuspended in supplemented RPMI-1640 media (Gibco™, Thermo Fisher Scientific Inc, Oxford, UK) containing 1% Penicillin-Streptomycin (Sigma-Aldrich Inc, Poole, UK); 1 % 2 mM L-glutamine (Gibco™, Thermo Fisher Scientific Inc, Oxford, UK) and 10 % Fetal Bovine Serum (Sigma-Aldrich Inc, Munich, Germany). PBMCs were stimulated with lipopolysaccharide (LPS) Escherichia coli (L4391, Sigma-Aldrich Inc, Munich, Germany) at 1 ng/ml or 5 ng/ml and cell culture supernatant was collected after 48 and 72 hours. All experiments were performed in duplicate and unstimulated cells acted as matched controls.

**9 DNA extraction and genotyping**

Genomic DNA was isolated from blood using QIAGEN® QIAamp DNA blood midi kit (QIAGEN®, Crawley, UK) according to manufacturer’s instructions. Up to 1 ml of red blood cell pellets were mixed with PBS to make a final volume of 2 ml. Samples were mixed with protease and incubated at 70 °C for 10 minutes. After mixing with ethanol the samples were loaded into QIAamp Midi columns and centrifuged. After to washing steps the DNA was eluted from the filter with dH_2_O. Optical density (OD) of the DNA solution was quantified at 260 nm and 280 nm using NanoDrop Spectophotometer and DNA concentration was measured with Qubit fluorimeter 3.0 using qubit broad range assay (Q32850, dsDNA Quantitation, broad range). Genomic DNA was stored at -20 °C until required.

Genotyping of the -460 and +405 VEGF-A single nucleotide polymorphisms (SNPs; genebank numbers rs833061 and rs2010963) ^5–7^ was performed using the SNP genotyping assay (Applied Biosystems, Foster City, CA)**.** Each assay contained 15 ng of genomic DNA, 12.5 µl of TaqMan Genotyping Master Mix, 1.25 µl 20 X SNP genotyping mix and DNAse free water in 25 µl of reaction volume. PCR and genotyping analysis was performed using 96-well plates (Armadillo PCR plate, semi-skirted, low profile clear wells, Thermo Fisher Scientific) on qPCR StepOnePlus machine. Standard thermal cycling conditions were used and consisted of an initial denaturation step at 95 ºC for 10 minutes followed by 40 cycles at 92ºC for 15 seconds (denature) and 60º for one minute (anneal/extension).

**References**

1. Tiirikainen ML, Woetmann A, Norsgaard H, Santamaria-Babí LF, Lovato P. Ex vivo culture of lesional psoriasis skin for pharmacological testing. J Dermatol Sci. 2020;97(2):109–16.

2. Luengas-Martínez A, Hardman-Smart J, Rutkowski D, Purba TS, Paus R, Young HS. Vascular endothelial growth factor blockade induces dermal endothelial cell apoptosis in a clinically relevant skin organ culture model. Skin Pharmacol Physiol. 2020;33:170–7.

3. Bankhead P, Loughrey MB, Fernández JA, Dombrowski Y, McArt DG, Dunne PD, et al. QuPath: Open source software for digital pathology image analysis. Sci Rep. 2017;7(1):16878.

4. Schindelin J, Arganda-Carreras I, Frise E, Kaynig V, Longair M, Pietzsch T, et al. Fiji: an open-source platform for biological-image analysis. Nat Methods. 2012;9(7):676–82.

5. Young HS, Bhushan M, Griffiths CEM, Summers AM, Brenchley PEC. Single-Nucleotide Polymorphisms of Vascular Endothelial Growth Factor in Psoriasis of Early Onset. J Invest Dermatol. 2004;122(1):209–15.

6. Young HS, Summers AM, Read IR, Fairhurst DA, Plant DJ, Campalani E, et al. Interaction between genetic control of vascular endothelial growth factor production and retinoid responsiveness in psoriasis. J Invest Dermatol. 2006;126(2):453–9.

7. Watson CJ, Webb NJA, Bottomley MJ, Brenchley PEC. Identification of polymorphisms within the vascular endothelial growth factor (VEGF) gene: Correlation with variation in VEGF protein production. Cytokine. 2000;12(8):1232–5.
