# Supplementary material for: Inhibition of vascular endothelial growth factor‐A downregulates angiogenesis in psoriasis: A pilot study
Source: Skin Health Dis. 2023 May 15;3(5):e245. doi: 10.1002/ski2.245 (PMC10549813; doi:10.1002/ski2.245)
Supplement: Supplementary file 2 — Supporting Information S2 [file SKI2-3-e245-s002.docx]

**Supplementary Tables**

**Table S1** Observed frequency of +405 VEGF-A gene polymorphism in the examined donors

|  | Healthy (n=5) | Psoriasis (n=6) | Severe psoriasis (n=3) | Mild/moderate psoriasis (n=3) |
| --- | --- | --- | --- | --- |
| Genotype |  |  |  |  |
| GG | 4 (80) | 2 (33.3) | 1 (33.3) | 1 (33.3) |
| GC | 0 | 3 (50) | 2 (66.6) | 1 (33.3) |
| CC | 1 (20) | 1 (16.6) | 0 | 1 (33.3) |
| Allele |  |  |  |  |
| G | 8 (80) | 7 (58.3) | 4 (66.6) | 3 (50) |
| C | 2 (20) | 5 (41.6) | 2 (33.3) | 3 (50) |

Data are presented as number (%).

**Table S2** Observed frequency of -460 VEGF-A gene polymorphism in the examined donors

|  | Healthy (n=5) | Psoriasis (n=6) | Severe psoriasis (n=3) | Mild/moderate psoriasis (n=3) |
| --- | --- | --- | --- | --- |
| Genotype |  |  |  |  |
| CC | 2 (40) | 2 (33.3) | 1 (33.3) | 1 (33.3) |
| CT | 2 (40) | 2 (33.3) | 2 (66.6) | 0 |
| TT | 1 (20) | 2 (33.3) | 0 | 2 (66.6) |
| Allele |  |  |  |  |
| C | 6 (60) | 6 (50) | 4 (66.6) | 2 (33.3) |
| T | 4 (40) | 6 (50) | 2 (33.3) | 4 (66.6) |

Data are presented as number (%).

**Supplementary Figures**

**
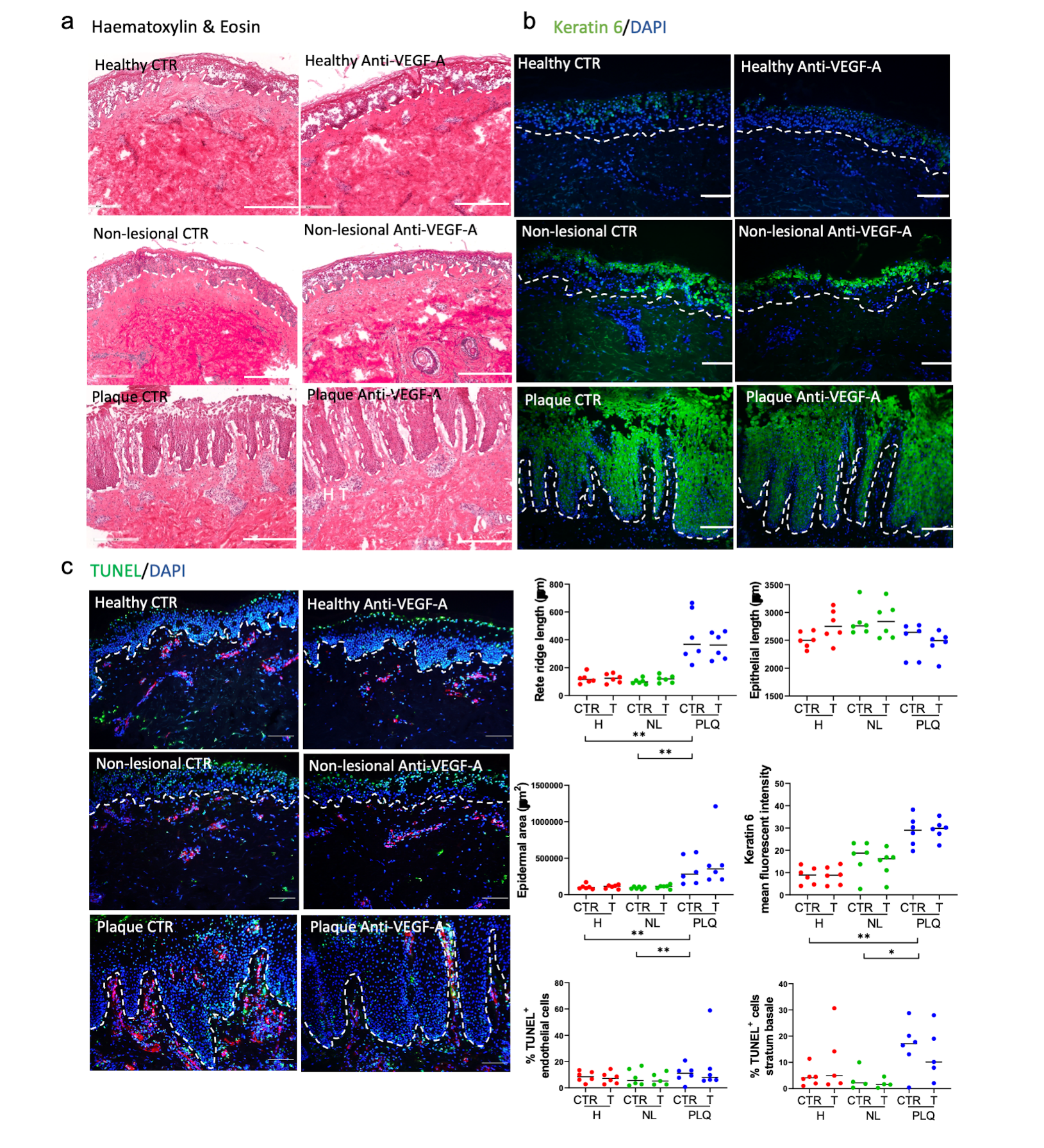
**

**Figure S1 Hematoxylin & Eosin stain, keratin 6 immunofluorescence stain and TUNEL assay**

a) Hematoxylin and eosin (H&E) was used to assess rete ridge length, epithelial length and epidermal area. The rete ridge length was greater in isotype control-treated plaque (367.8 [360.8] µm) compared to isotype control-treated non-lesional (97.76 [27.85] µm; ***p*<0.01) and isotype control-treated healthy skin (116 [48.2] µm; ***p*<0.01). The epidermal area in isotype control-treated plaque (281485 [406360] µm^2^) was higher than in isotype control-treated non-lesional (93793 [29350] µm^2^; ***p*<0.01) and isotype control-treated healthy (97238 [42165] µm^2^; ***p*<0.01). b) Keratin 6 fluorescent intensity, assessed in the epidermis, was higher in isotype control-treated plaques (29.02 [12.14]) compared to isotype control-treated non-lesional (18.76 [12.23]; **p*<0.05) and isotype control-treated healthy skin (8.92 [7.78]; ***p*<0.01). c) VEGF-A inhibition did not affect TUNEL expression in the stratum basale or in endothelial cells. Number of independent experiments n=36 (1 punch biopsy per patient and per treatment group). Data were presented as median and were analysed with two-tailed Wilcoxon matched-pairs signed rank test (paired analysis) or two-tailed Mann Whitney test (unpaired analysis). Scale bar = 100 µm, except from H&E, where scale bars = 300 µm. * *p <* 0.05, ** *p <* 0.01. CTR: isotype control; H: healthy; NL: non-lesional; PLQ: plaque; T: treated with anti-VEGF-A monoclonal antibody.

**
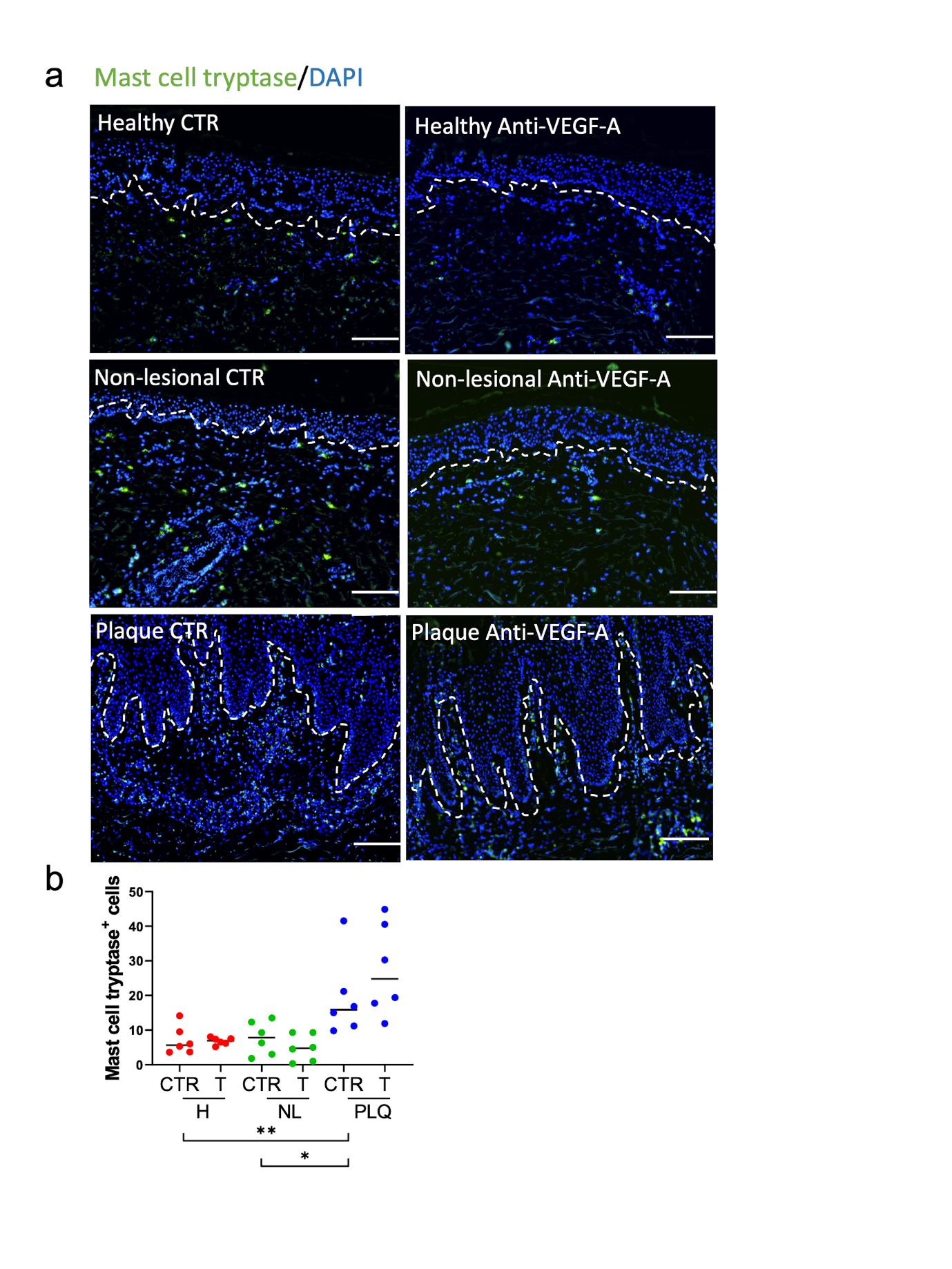
**

**Figure S2 Tryptase^+^-mast cell expression remained unaltered in ECs and the stratum basale**

a) The number of tryptase^+^ mast cell was assessed in the dermis of healthy, non-lesional and plaque skin. DAPI stained the nuclei. b) Tryptase^+^ mast cell number was higher in isotype control-treated plaque (15.92 [15.47]) than in isotype control-treated non-lesional (7.81 [9.9]; **p*<0.05) and isotype control-treated healthy (5.667 [7.01]; ***p*<0.01). Number of independent experiments n=36 (1 punch biopsy per patient and per treatment group). Data were presented as median and were analysed with two-tailed Wilcoxon matched-pairs signed rank test (paired analysis) or two-tailed Mann Whitney test (unpaired analysis). Scale bars = 100µm. * *p <* 0.05, ** *p <* 0.01. CTR: isotype control; H: healthy; NL: non-lesional; PLQ: plaque; T: treated with anti-VEGF-A monoclonal antibody.

**
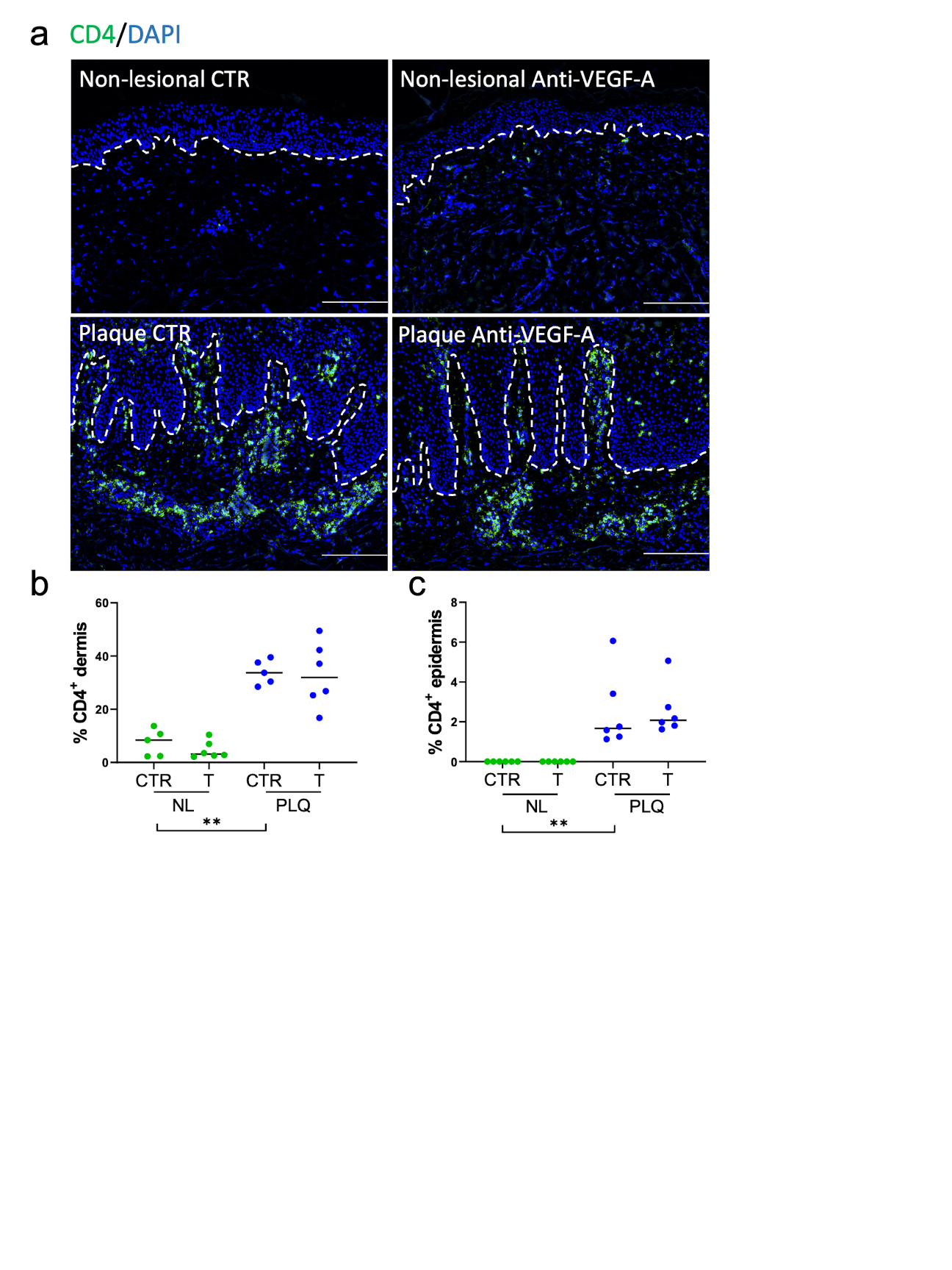
**

**Figure S3 VEGF-A inhibition did not alter CD4^+^ expression**

a) The number of CD4^+^ cells was assessed in the dermis superficial to the deep vascular plexus and in the epidermis of non-lesional and plaque skin samples. DAPI stained the nuclei. b) In the dermis, the number of CD4^+^ cells was higher in isotype control-treated plaque [33.71 (9.11)] than in isotype control-treated non-lesional [8.43 (9.85); ***p*<0.01]. c) In the epidermis, the number of CD4^+^ cells was higher in isotype control-treated plaques [1.67 (2.84)] than in isotype control-treated non-lesional [0 (0); ***p*<0.01]. Number of independent experiments n=24 (1 punch biopsy per patient and per treatment group). Data were presented as median and were analysed with two-tailed Wilcoxon matched-pairs signed rank test (paired analysis) or two-tailed Mann Whitney test (unpaired analysis). Scale bars = 200 µm. *p <* 0.05, ** *p <* 0.01. CTR: isotype control; H: healthy; NL: non-lesional; PLQ: plaque; T: treated with anti-VEGF-A monoclonal antibody.


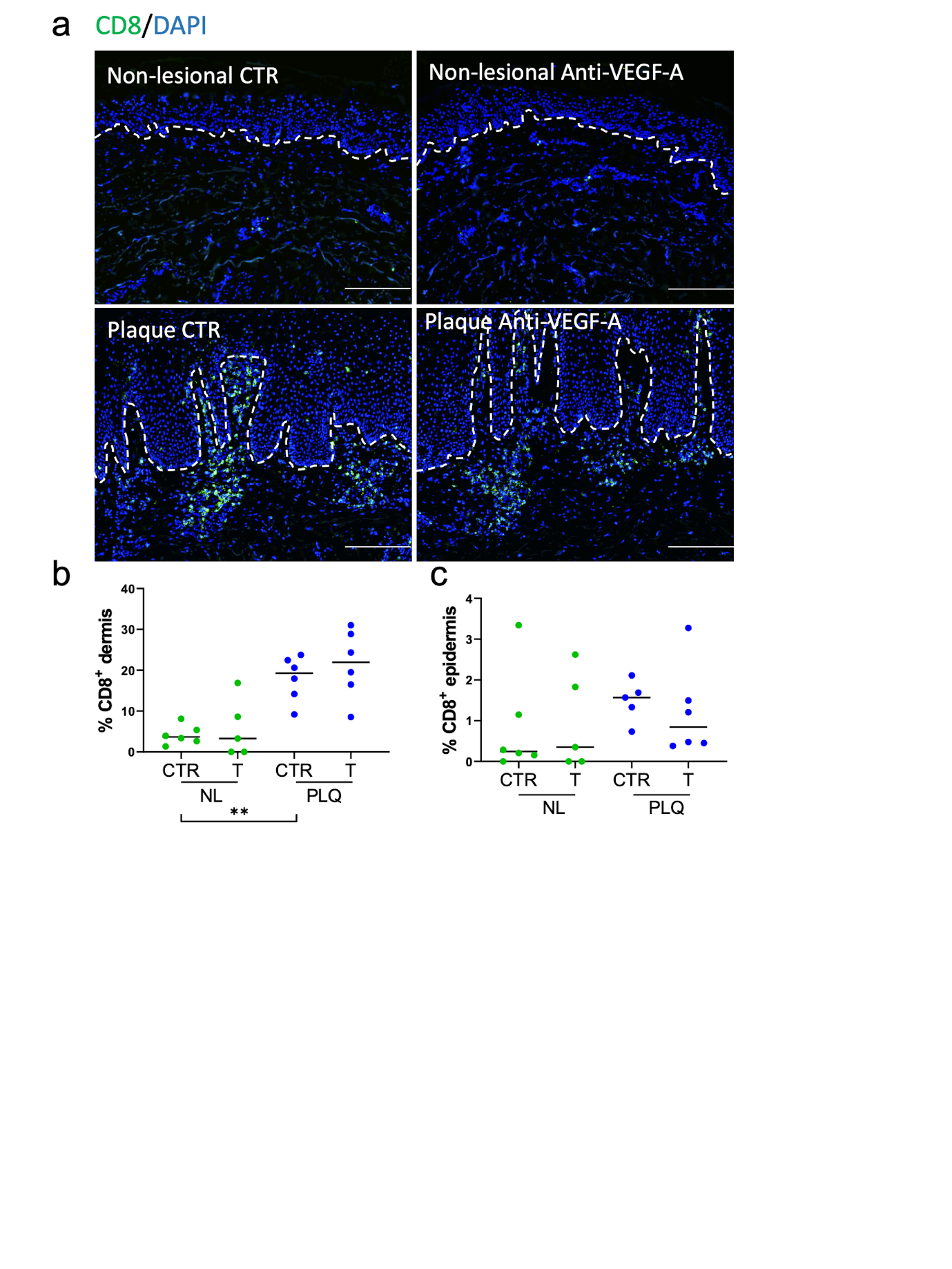


**Figure S4 VEGF-A inhibition did not alter CD8^+^ expression**

The number of CD8^+^ cells was assessed in the dermis superficial to the deep vascular plexus and in the epidermis of non-lesional and plaque skin samples. DAPI stained the nuclei. In the dermis, the number of CD8^+^ cells was higher in isotype control-treated plaques (19.28 [9.86]) than in isotype control-treated non-lesional (3.67 [3.72]; ***p*<0.01). Number of independent experiments n=24 (1 punch biopsy per patient and per treatment group). Data were presented as median and were analysed with two-tailed Wilcoxon matched-pairs signed rank test (paired analysis) or two-tailed Mann Whitney test (unpaired analysis). Scale bars = 200 µm. ** *p <* 0.01. CTR: isotype control; H: healthy; NL: non-lesional; PLQ: plaque; T: treated with anti-VEGF-A monoclonal antibody.

**
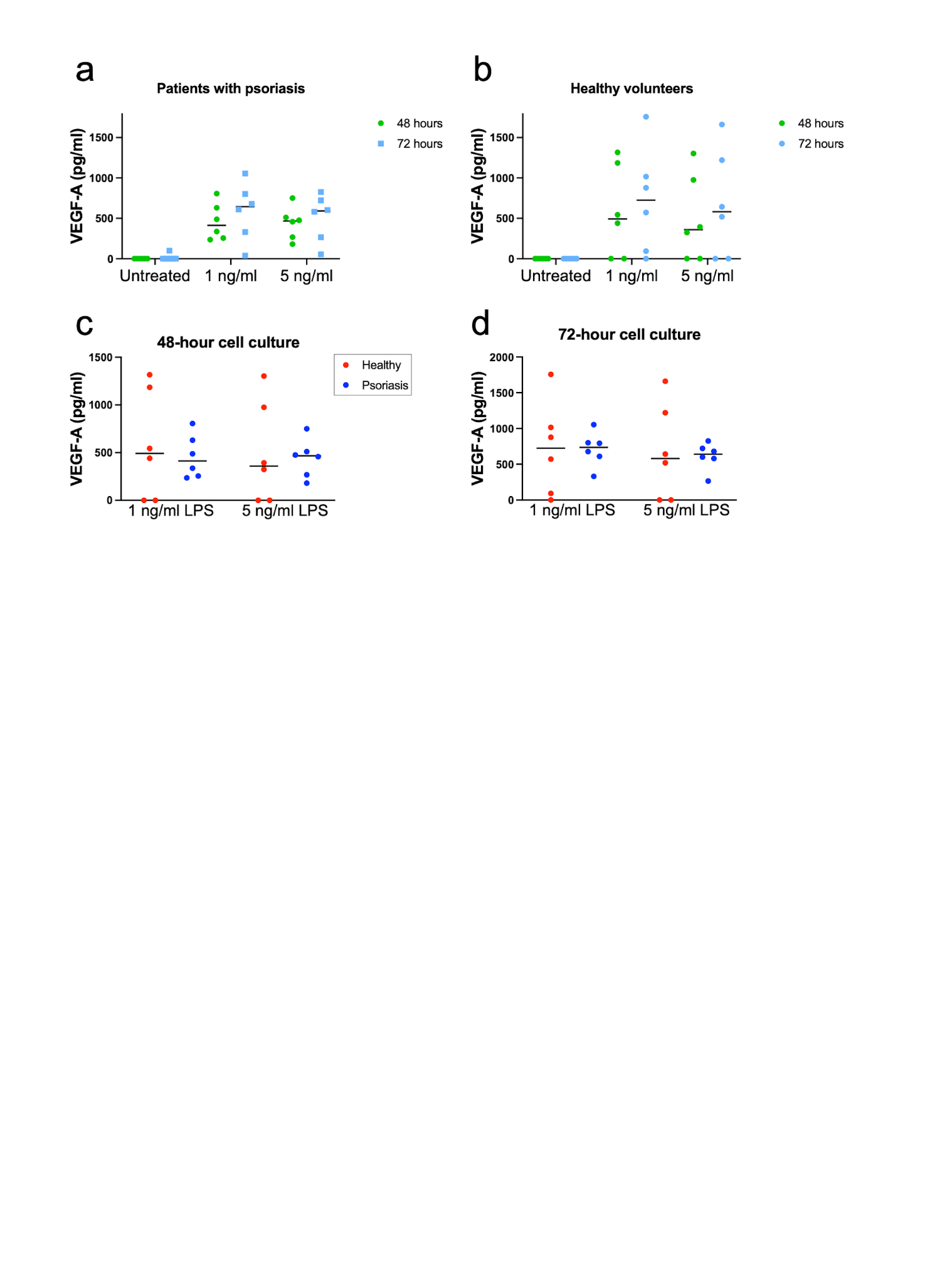
**

**Figure S5 VEGF-A production by peripheral blood mononuclear cells *in vitro***

a) Peripheral blood mononuclear cells (PBMCs) from patients with psoriasis and b) healthy volunteers were incubated with 1 ng/ml of lipopolysaccharide (LPS) or 5 ng/ml of LPS for 48 or 72 hours. Untreated cells were used as controls. There were not significant differences between the levels of VEGF-A produced by PBMCs of healthy volunteers and patients with psoriasis at c) 48 hours and d) 72 hours. Data were analysed with two-tailed Mann Whitney test for unpaired analysis.
